# Inhibition of IκB Kinase by Vaccinia Virus Virulence Factor B14

**DOI:** 10.1371/journal.ppat.0040022

**Published:** 2008-02-08

**Authors:** Ron A.-J Chen, Grigory Ryzhakov, Samantha Cooray, Felix Randow, Geoffrey L Smith

**Affiliations:** 1 Department of Virology, Faculty of Medicine, Imperial College London, London, United Kingdom; 2 Medical Research Council Laboratory of Molecular Biology, Cambridge, United Kingdom; Saint Louis University, United States of America

## Abstract

The IκB kinase (IKK) complex is a key regulator of signal transduction pathways leading to the induction of NF-κB-dependent gene expression and production of pro-inflammatory cytokines. It therefore represents a major target for the development of anti-inflammatory therapeutic drugs and may be targeted by pathogens seeking to diminish the host response to infection. Previously, the vaccinia virus (VACV) strain Western Reserve B14 protein was characterised as an intracellular virulence factor that alters the inflammatory response to infection by an unknown mechanism. Here we demonstrate that ectopic expression of B14 inhibited NF-κB activation in response to TNFα, IL-1β, poly(I:C), and PMA. In cells infected with VACV lacking gene *B14R* (vΔB14) there was a higher level of phosphorylated IκBα but a similar level of IκBα compared to cells infected with control viruses expressing B14, suggesting B14 affects IKK activity. Direct evidence for this was obtained by showing that B14 co-purified and co-precipitated with the endogenous IKK complex from human and mouse cells and inhibited IKK complex enzymatic activity. Notably, the interaction between B14 and the IKK complex required IKKβ but not IKKα, suggesting the interaction occurs via IKKβ. B14 inhibited NF-κB activation induced by overexpression of IKKα, IKKβ, and a constitutively active mutant of IKKα, S176/180E, but did not inhibit a comparable mutant of IKKβ, S177/181E. This suggested that phosphorylation of these serine residues in the activation loop of IKKβ is targeted by B14, and this was confirmed using Ab specific for phospho-IKKβ.

## Author Summary

Vaccinia virus (VACV) is the live vaccine used to eradicate smallpox and is also the most intensively studied poxvirus. Like many poxviruses, VACV produces a wide variety of proteins that inhibit parts of the host response to infection. Consequently, the virus can escape destruction by the immune system and be passed on to additional hosts. Here we report a new VACV immune evasion mechanism mediated by protein B14, a protein that contributes to virus virulence. B14 functions by interacting with a cellular protein called IKKβ, which is critical for mounting an innate immune response to infection, and also plays important roles in cancer and cell death. B14 prevents IKKβ being activated and consequently the cellular signaling pathway leading to activation of nuclear factor kappa B (NF-κB) is not induced. Without activation of NF-κB the host cell cannot produce other molecules that amplify the innate immune response to infection. This mechanism of action of B14 fits nicely with the observed increase in the host response to infection by a VACV strain lacking the *B14R* gene. Lastly, an increased understanding of how B14 inhibits IKKβ function may lead to development of novel drugs against this important cellular enzyme.

## Introduction

Nuclear factor-κB (NF-κB) is critical for the innate and adaptive immune responses to infection. Various stimuli, such as the pro-inflammatory cytokines interleukin (IL)-1 and tumour necrosis factor (TNF), activate signaling pathways leading to NF-κB-dependent gene expression [1,2]. Several of these signaling pathways converge on the IKK complex [3–5], and this complex is therefore a prime target for anti-inflammatory drugs. It is also a logical target for pathogens aiming to minimize the host response to infection. The IKK complex, or signalosome, comprises a heterodimer of IKKα and IKKβ in association with NF-κB essential modifier (NEMO also called IKKγ) [6,7] and is critical for NF-κB activation induced by pro-inflammatory cytokines [8–10]. The IKK complex is activated by upstream kinases, such as transforming growth factor-β (TGFβ)-activated kinase-1 (TAK1), which phosphorylates IKKβ at Ser177 and Ser181 located in the activation loop [2,4,5]. Once activated, IKKβ phosphorylates the inhibitor of NF-κB (IκBα) [11] to initiate IκBα degradation. Phosphorylated IκBα (phospho-IκBα) is recognized by an F-box/WD protein, β-transducin repeats-containing proteins (β-TrCP), which functions as a receptor subunit of the SCF family ubiquitin ligase complex, and binds to the phosphorylated E3 recognition sequence on IκBα [12–15]. This poly-ubiquitinated IκBα remains associated with NF-κB but is degraded selectively via the 26S proteasome [16]. After IκBα degradation, NF-κB is translocated into the nucleus to induce transcription of responsive genes [17].

Poxviruses have developed strategies to modulate important cellular signaling pathways to evade host responses [18–20]. These viruses target many of the primary mediators of immune system including IL-1, IL-18, interferons (IFNs), TNF, complement, and chemokines [20–23]. Many of the genes encoding vaccinia virus (VACV) immunomodulators show amino acid similarity to host proteins that function in the immune system. However, others lack such similarity; for instance, the intracellular virulence factor N1 [24], anti-apoptotic protein F1 [25,26], and secreted chemokine binding protein [27].

VACV and other poxviruses interrupt the activity of NF-κB in several ways [21,28]. One strategy is to secrete proteins from the infected cell to bind cytokines, chemokines, or IFNs and prevent these reaching their receptors on cells. Another strategy is to express intracellular factors to regulate signaling pathways leading to NF-κB activation. Among these intracellular inhibitors, VACV proteins A52 and A46 antagonize IL-1R and toll like receptor (TLR) signaling [29–31] and N1 is a virulence factor [24] that is reported to interfere with NF-κB and IRF3 activity [32]. In addition, the crystal structure of N1 reveals it is a Bcl-2-like protein and N1 was shown to protect cells from apoptosis [33]. VACV protein K1 also inhibits NF-κB activation during infection [34]. Lastly, protein M2 downregulates ERK-mediated NF-κB induction in virus-infected cells [35].

*B14R* is one of the few VACV genes that are located in the terminal region of the virus genome and yet is conserved in many orthopoxviruses [36], suggesting an important function. However, B14 lacks sequence identity with proteins from outside poxviruses. An initial characterization of B14 showed it is an intracellular virulence factor that is expressed early during infection and affects the inflammatory response to infection in a murine model by an unknown mechanism [37]. In this study, the mechanism of action of B14 has been investigated. Data presented show that B14 associates with and inhibits the activity of the IKK complex and thereby inhibits NF-κB activation from multiple signaling pathways. This mechanism of action is consistent with the in vivo phenotype of a virus lacking the *B14R* gene [37].

## Results

### B14 Inhibits Multiple Signaling Pathways Leading to NF-κB Activation

Bioinformatic analyses indicated that B14 is a member of a family of poxvirus proteins that include B14, K7, C6, and A52 [38]. Subsequently, the A46 protein was shown to be related to A52 and was added to this family [29]. Given that proteins A46 and A52 are intracellular inhibitors of TLR signaling pathways [29–31], the presence of B14 in the same family suggested that B14 might also act to regulate signaling pathways leading to NF-κB activation.

**Figure 1:**
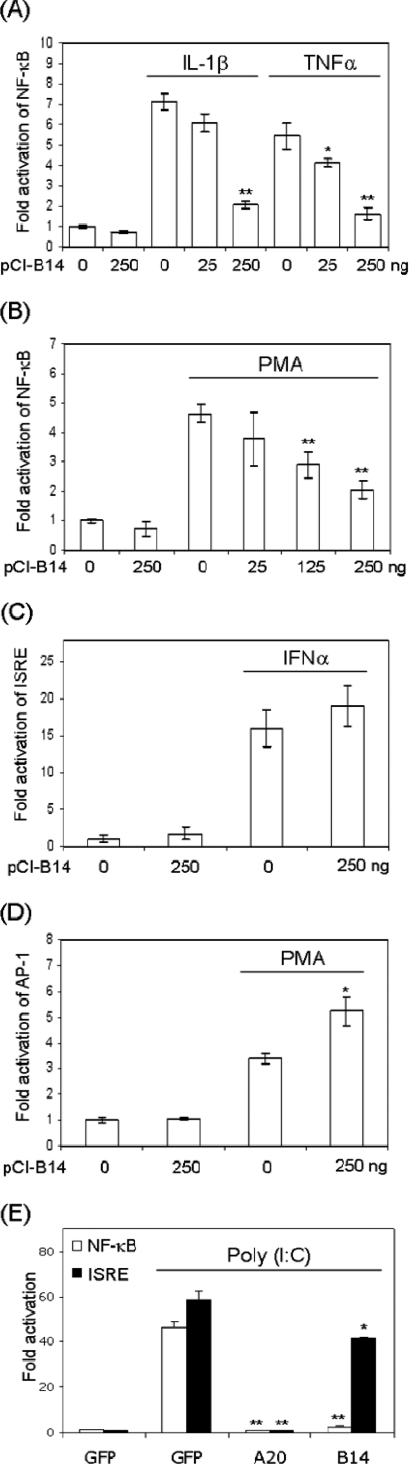
B14 Inhibits NF-κB Activation HeLa cells were co-transfected with reporter plasmids for 100 ng of NF-κB (A and B), ISRE (C), AP-1 (D), and 50 ng of pSV-β-galactosidase in all cases. pCI-B14 was transfected where indicated and the amount (ng) per well is shown. The total amount of DNA applied in each reaction was adjusted to 400 ng using pCI empty vector. These transfected cells were stimulated with 100 ng/ml of IL-1β, TNFα, IFNα, or 50 ng/ml of PMA for 8 h as indicated. Luciferase activity was measured and normalized to β-galactosidase intensity in the same well in triplicate wells. (E) HEK 293 cells were co-transfected with pRL-TK and reporter plasmid for NF-κB (□), ISRE (▪), and plasmids expressing GFP, A20, and B14. These cells were stimulated with 5 μg/ml of poly(I:C) for 12 h and then were lysed to measure luciferase activity using Dual-specific luciferase assay kit (Promega). Differences in the fold activation of the indicated reporter in cells transfected with the empty vector and cells expressing B14 were analysed by Student's *t*-test and the *p*-values are indicated: * *p* < 0.05 or ** *p* < 0.01.

To investigate the effect of B14 on NF-κB activation, a plasmid containing a luciferase reporter gene linked to a NF-κB-dependent promoter was transfected into HeLa cells and these cells were stimulated with IL-1β (Figure 1A), TNFα (Figure 1A), or PMA (Figure 1B). Luciferase activity was increased greatly by addition of each stimulant but the level reached was reduced in a dose-dependent manner in the presence of B14. Similar findings were observed using HEK 293 cells (unpublished data). Moreover, B14 decreased poly (I:C)-induced NF-κB dramatically (*p-*value = 0.0006; 95% decrease) (Figure 1E). In contrast, B14 did not reduce luciferase activity from ISRE (Figure 1C) and AP-1 (Figure 1D) reporter genes induced by IFNα and PMA, respectively. Notably, B14 increased PMA-induced AP-1 activity slightly (*p* = 0.02; 1.5-fold increase; Figure 1D). We also observed a small but significant reduction in poly (I:C)-induced ISRE activity in the presence of B14 (*p-*value = 0.01; 29% decrease; Figure 1E). However, it is uncertain if these relatively small changes seen with these reporter assays are relevant biologically. As a control we also expressed A20, a de-ubiquitinating enzyme that downregulates NF-κB and IRF3 [39–41] and observed strong inhibition of both pathways (Figure 1E).

Therefore, B14 is a specific downregulator of NF-κB but did not inhibit AP-1 or IRF responsive gene expression. The fact that B14 inhibits multiple pathways leading to NF-κB activation suggests that B14 might act at a position at or downstream of the site at which these pathways converge, namely the IKK complex.

### B14 Associates with the IKK Signalosome

**Figure 2:**
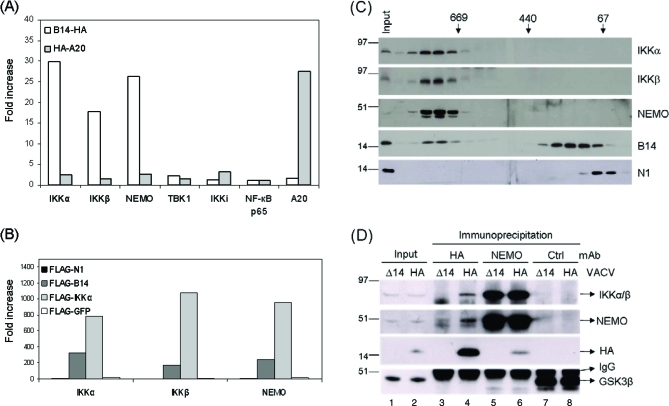
VACV-Expressed B14 Associates with the IKK Signaling Complex Cells were co-transfected with expression vectors for indicated proteins tagged with HA (A) or FLAG (B) epitopes and the indicated protein fused with luciferase. The indicated tagged bait protein was immunoprecipated by correspondent monoclonal Ab and then the immune complex was eluted to be analysed for luciferase activity. Data presented are from one of the three independent experiments. (C) HeLa cells were infected with vB14 and a cell extract was prepared and analysed by size exclusion chromatography on a Superose 6 column. An aliquot of each fraction was separated in 4%–12% NuPAGE and analysed by immunoblotting with the indicated Abs. The position of protein size markers is indicated in kDa at the top. (D) Extracts from HeLa cells infected with the vΔB14 (Δ) or vB14-HA (HA) were pre-cleared and immunoprecipitated with mAbs against HA, NEMO, and GSK3β (isotype control). The immunoprecipitated proteins were resolved in 4%–12% NuPAGE and then were analysed by immunoblotting with the Abs indicated on the right, respectively. Sizes of bands detected are indicated on the left in kDa.

To examine how B14 downregulates NF-κB activation, we searched for interactions between B14 and potential ligands using a luminescence-based mammalian interactome mapping (LUMIER) assay [42]. Components of the IKK complex were included in the assay because several pathways leading to NF-κB activation converge on this complex. HA-tagged B14 and A20 were transfected into cells together with different proteins fused with luciferase, and cell extracts were immunoprecipitated with anti-HA mAb. The immunoprecipitates were then tested for luciferase activity (Figure 2A). B14 interacted with IKKα, IKKβ, and NEMO but not with TBK1, IKKɛ, p65, and A20. As expected, A20 showed no interaction with any proteins screened in the assay except itself [43]. These observations indicated that B14 interacts with the IKK complex. Previously, VACV protein N1 was reported to interact with and inhibit the IKK complex [32] and therefore FLAG-tagged N1 was also included in this assay. Surprisingly, no interaction between N1 and IKKα, IKKβ, or NEMO was observed (Figure 2B), although FLAG-B14 and IKKα each co-precipitated with the IKKs. FLAG-GFP was included as a negative control and did not bind to the IKKs. Collectively, these data show that B14, but not N1, associates with the IKK signalosome.

To investigate these protein interactions further, we fractionated VACV-infected cell extracts by size exclusion chromatography (SEC) and blotted the fractions with antibody to B14. B14 eluted in two peaks corresponding to approximately 160 kDa and 700–900 kDa, despite having a monomeric size of 17 kDa. Given that the IKK complex also has a mass of between 700–900 kDa [1], we immunoblotted the column fractions with antibodies to IKK components and found that the IKK complex co-purified with the first B14 peak (Figure 2C). The column fractions were also blotted with Ab to N1 and this showed that N1 eluted with a mass of approximately 60–70 kDa (Figure 2C), quite distinct from the IKK complex and also distinct from the expected position of the 28-kDa N1 homo-dimer [24]. Therefore, B14, but not N1, co-purified with the IKK complex in the VACV-infected cell lysates.

The possible interaction between B14 and the IKK complex was investigated further by immunoprecipitation. HeLa cells were infected with a VACV strain expressing an HA-tagged version of B14 (vB14-HA) or VACV lacking gene *B14R* (vΔB14) [37], and cytoplasmic extracts were prepared. B14-HA was immunoprecipitated with anti-HA mAb and immunoprecipitates were analysed by immunoblotting with Abs against IKKα and IKKβ, NEMO, or HA (Figure 2D). The anti-HA mAb precipitated B14-HA together with IKKα, IKKβ, and NEMO from the vB14-HA infected cell lysates (Figure 2D, lane 4). The interaction between B14 and the IKK complex was also seen in the reciprocal immunoprecipitation using antibody to NEMO (Figure 2D, lanes 5 and 6) and anti-IKKα/β (unpublished data). In contrast, B14 and the IKK complex were not co-immunoprecipitated with a control mAb against glycogen synthase kinase (GSK)-3β (Figure 2D, lanes 7 and 8). In summary, B14 and the IKK complex co-purified and co-precipitated when each component was expressed at natural levels.

**Figure 3:**
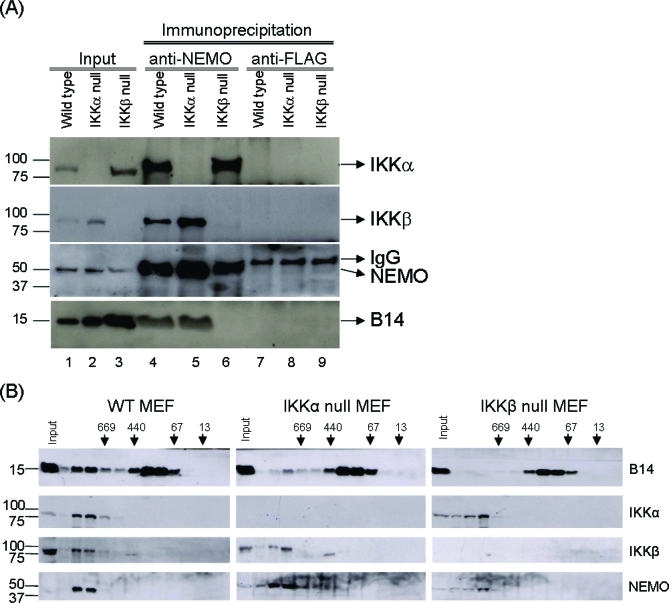
B14 Binds to the IKK Complex via IKKβ (A) Wild type, IKKα null, and IKKβ null MEF cells were infected with vB14-HA and cell extracts were immunoprecipitated using anti-NEMO mAb. The immunocomplex was resolved by SDS-PAGE (12% gel) and analysed by immunoblotting using the indicated Abs. (B) Cell extracts from vB14-HA-infected MEFs were prepared and analysed by SEC on a Superose 6 column. Proteins from each fraction were precipitated using trichloroacetic acid and 15% of the sample was separated in a 12% SDS-PAGE and analysed by immunoblotting using the indicated Abs.

To identify which of the IKK components interacts with B14, mouse embryo fibroblasts (MEFs) lacking IKKα or IKKβ were analysed as above for HeLa cells (Figure 3). In vB14-HA-infected wild type MEFs B14 co-precipitated with the IKK complex (Figure 3A, lane 4). In the absence of IKKα or IKKβ, the anti-NEMO mAb still precipitated a complex of IKKβ-NEMO and IKKα-NEMO, respectively (lanes 5 and 6). However, B14 was co-precipitated from IKKα but not IKKβ null MEFs, indicating that B14 was incorporated in the IKKβ-NEMO complex (lane 5) and that IKKβ was needed for B14 to be part of the IKK complex. As a control, an anti-FLAG mAb did not immunoprecipitate any proteins (Figure 3A, lanes 7–9). The interaction between B14 and IKKβ was also investigated by SEC of extracts from wild type, IKKα, or IKKβ null MEFs (Figure 3B). B14 only co-purified with the IKK complex of 700–900 kDa when IKKβ was present, but was present in the second peak of 160 kDa in all samples. So, IKKβ is necessary for B14 to co-purify or co-precipitate with the IKK complex.

### IKK Activity Is Downregulated by B14

**Figure 4:**
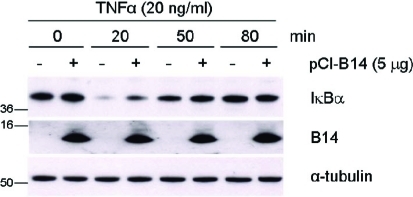
The Effect of B14 on IκBα B14 was expressed in HeLa cells by transfection and the cells were treated with TNFα for the indicated times. Then TNF was washed away and extracts were prepared and analysed by immunoblotting with the indicated Abs. Cell lysates (15 μg per lane) were resolved by SDS-PAGE (15% gel) and analysed by immunoblotting with the Abs indicated on the right. Sizes of bands detected are indicated on the left in kDa.

Upon stimulation, the IKK complex phosphorylates IκBα and this is then removed quickly via the proteasome system. Therefore, we examined the level of IκBα in cells stimulated with TNFα in the presence and absence of B14 (Figure 4). The amount of IκBα was reduced dramatically at 20 min after TNF treatment but had recovered to the original level by 50 min. However, in the presence of B14 the level of IκBα was greater at 20 min post-stimulation with TNF. Thereafter, the level of IκBα recovered to that before stimulation. Equal loading of samples was demonstrated by blotting for α-tubulin. Therefore, B14 increased IκBα stability after TNF stimulation, implying a negative effect on IKK activity.

**Figure 5:**
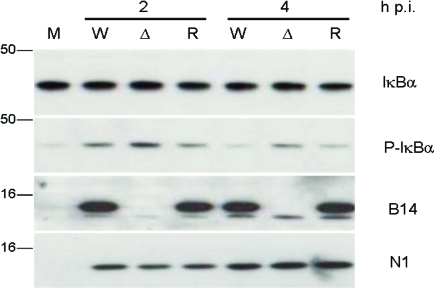
B14 Reduces the Phosphorylation of IκBα in VACV-Infected Cells Cytoplasmic extracts were prepared from HeLa cells at the indicated times after mock-infection (M) or infection with vB14 (W), vΔB14(Δ), and vB14-rev (R) at 2 p.f.u. per cell. Cytosolic proteins (15 μg per lane) were resolved by SDS-PAGE (15% gel) and analysed by immunoblotting with the Abs indicated on the right. Sizes of bands detected are indicated on the left in kDa.

The above experiment was performed in cells expressing B14 after transfection. To investigate whether the endogenous levels of B14 could affect IKK activity during virus infection, the phosphorylation status of IκBα was investigated in cells infected with VACV strains that do or do not express B14. Cells were infected with wild type (vB14), deletion mutant (vΔB14), or revertant (vB14-rev) viruses [37] at 2 p.f.u./cell and at 2 and 4 h p.i., cytoplasmic fractions were prepared and analysed by immunoblotting (Figure 5). The level of IκBα was indistinguishable in infected or uninfected cells, and similarly there was no difference following infection with viruses that did or did not express B14. However, following infection by all viruses, the level of phospho-IκBα was increased, but the increase was noticeably higher in cells infected with vΔB14, compared to vB14 and vB14-rev. To show that each virus caused equivalent infection, cell extracts were immunoblotted with antibody to the VACV intracellular protein N1 [24], and N1 was detected at similar levels in each sample at 2 h p.i. and at slightly higher levels in each sample later during infection (4 h) (Figure 5, bottom panel, lanes 2–4 and 6–8). In contrast, B14 was present in vB14- and vB14-rev-infected cells only. As expected, each VACV protein was absent in mock-infected cells. This suggested that B14 reduces IKK activity during VACV infection.

**Figure 6:**
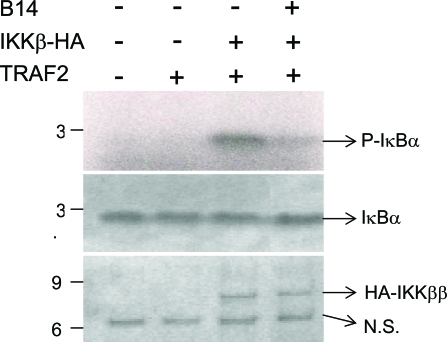
B14 Inhibits IKK-Mediated Phosphorylation of IκBα HEK 293 cells were transfected with plasmids expressing the indicated proteins (2 μg of vectors expressing B14, TRAF2, and HA-IKKβ). The empty vector pCI was used to adjust the total DNA added to 6 μg per reaction per 10 cm dish. After 24 h, 1.5 mg of cell extracts were immunoprecipitated with anti-HA mAb for 3 h at 4 °C. The immune complex was incubated with 5 μg of IκBα peptide and 5 μCi ^32^PγATP for 10 min at 30 °C. The kinase assay was terminated by adding protein loading buffer and the mixture was resolved and analysed by Coomassie staining followed by autoradiography (top panel, kinase assay). Protein markers (kDa) were indicated on the left. N.S. indicates non-specific band.

The effect of B14 on IKK activity in the absence of other VACV-encoded NF-κB inhibitors was investigated next using an in vitro kinase assay. Plasmids expressing TRAF2 or HA-tagged IKKβ were co-transfected with or without pCI-B14. TRAF2 acts as an intracellular stimulator of IKK activity. Extracts from transfected cells were immunoprecipitated with anti-HA mAb. The activity of the immunoprecipitated IKK complex was studied using a synthetic IκBα peptide substrate and ^32^P-γ-ATP followed by SDS-PAGE and autoradiography. Notably, the level of the phospho-IκBα peptide was reduced in the presence of B14, indicating B14 inhibited IKK activity (Figure 6). Coomassie blue staining of the SDS-polyacrylamide gel indicated that similar amount of the immunoprecipitated HA-IKKβ and substrate peptides were applied in the assay (Figure 6, lower panels).

**Figure 7:**
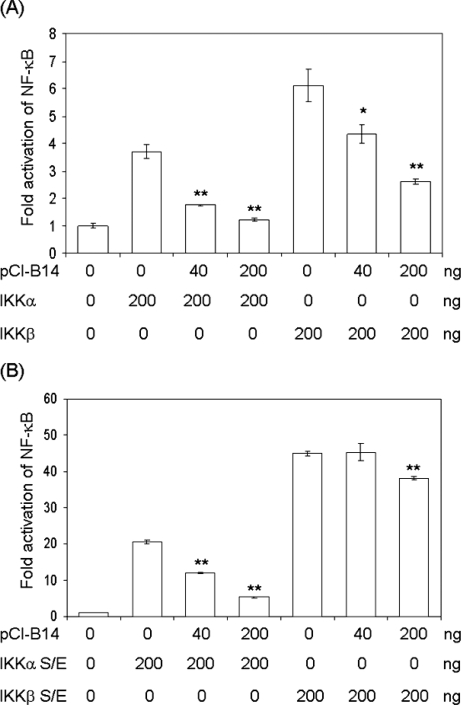
The Effect of B14 on IKKα and IKKβ-Induced NF-κB Activation HEK 293 cells were co-transfected with reporter plasmids for NF-κB (90 ng), TK-RhL as internal control (10 ng), and the indicated amount of plasmids expressing either wild type IKKα or IKKβ (A), constitutively active IKKα or IKKβ (B), or B14 (A and B). The total amount of DNA applied in each reaction was adjusted to 500 ng using pCI empty vector. After 24 h, the cells were lysed and the activities of both luciferases were measured using Dual-specific luciferase assay kit (Promega) in triplicates. Differences in the fold activation of the NF-κB reporter induced by the indicated IKK subunit in cells transfected with the empty vector and cells expressing B14 were analysed by Student's *t*-test and the *p-*values are indicated: * *p* < 0.05 or ** *p* < 0.01.

To study the effect of B14 on IKK activity further, the IKK complex was activated by overexpression of either IKKα or IKKβ (Figure 7A), and B14 was found to inhibit this activation significantly and in a dose-dependent manner (Figure 7A). This indicated that B14 acts at, or downstream of, the IKK signalosome. The site of action was investigated further using IKK constitutively active mutants, IKKα SS/EE and IKKβ SS/EE that contain mutations in the activation loop [2] (Figure 7B). B14 inhibited IKKα SS/EE significantly and in a dose-dependent manner. In contrast, there was only a small (15%) reduction of IKKβ SS/EE-induced NF-κB activation in the presence of the highest amount of B14. These findings imply that once IKKβ is activated, B14 can no longer prevent NF-κB activation and also suggest a model in which B14 inhibits activation of the IKK complex by preventing phosphorylation of IKKβ in the activation loop.

**Figure 8:**
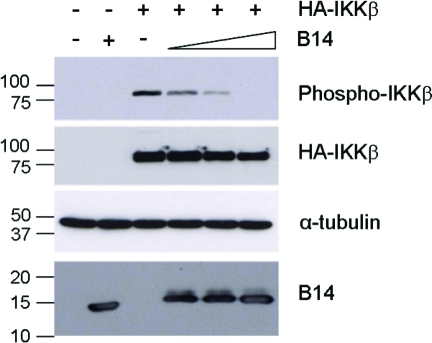
B14 Inhibits Phosphorylation of IKKβ on Ser 177/181 HEK 293T cells were transfected with vectors expressing B14 (3, 1.2, and 0.6 μg) and HA-IKKβ (2 μg). The empty vector pCI was used to adjust the total DNA added to 5 μg per reaction per 6 cm dish. After 24 h, cell lysates were resolved by SDS-PAGE (15% gel) and analysed by immunoblotting using the Abs indicated on the right (the anti-HA mAb was used to detect total HA-IKKβ). Sizes of bands detected are indicated on the left in kDa.

This hypothesis was tested directly by using Ab to detect IKKβ that has been phosphorylated in the activation loop at serine 177 and 181 (Figure 8). HA-tagged IKKβ was transfected into 293 T cells either alone or together with increasing concentrations of B14. In the absence of transfected HA-IKKβ no phospho-IKKβ was detected, but after addition of HA-IKKβ, phospho-IKKβ was observed easily and was reduced in a dose-dependent manner as the concentration of B14 increased. Notably, while the amount of phospho-IKKβ decreased in the presence of B14, the amount of total HA-IKKβ remained fairly constant and blotting for tubulin confirmed equal loading of samples. Therefore, B14 inhibits NF-κB activation by preventing phosphorylation of IKKβ in the activation loop.

##  Discussion

In this study, VACV protein B14 is shown to inhibit the IKK complex and to downregulate NF-κB-dependent gene expression, which is crucial for the innate and adaptive immune response to infection [3,4]. Our previous in vivo study, using recombinant VACVs that do or do not express B14, demonstrated B14 is an intracellular virulence factor that modulates the inflammatory response in vivo [37]. The activity of B14 described here is consistent with this phenotype: downregulation of NF-κB-dependent expression of pro-inflammatory cytokines will alter recruitment of inflammatory cells to sites of infection and so diminish the ability of the host to fight infection. Notably, a virus lacking the *B14R* gene was attenuated compared to parental virus [37].

The IKK complex is critical for activation of NF-κB [3,44–46] and therefore is a logical target for modulation by pathogens [4,47]. B14 is one of several VACV proteins that inhibit signaling pathways leading to NF-κB activation, but these proteins all have non-redundant functions because when the gene encoding each inhibitor is deleted individually, the deletion mutant displays an in vivo phenotype [24,29–31,37]. Therefore, these proteins must each have distinct functions. In this regard, B14 differs from A46 and A52 in that it targets a broader array of immune signaling pathways; for instance, A46 and A52 inhibit IL-1 but not TNF-induced signaling, whereas B14 inhibits both (Figure 1A). Also A46 and A52 target the signaling pathways upstream of the IKK complex [29–31], whereas B14 targets the activity of the IKK complex. B14 also differs from N1 in that N1 was reported to inhibit signaling pathways leading to NF-κB activation [32] and to IFN responses via TBK1 [32], whereas B14 did not inhibit IFN responses induced by either IFNα or poly (I:C) (Figure 1E). N1 was reported to target to the IKK signalosome by binding to the kinase complex when both components were overexpressed [32]. However, three independent experiments shown here contradict this: first, N1 did not bind to IKK components in the LUMIER assay (Figure 2B); second, N1 did not co-purify with IKK during biochemical fractionation of infected cells (Figure 2C); and third, N1 did not co-precipitate with IKK components using the anti-NEMO mAb (unpublished data). In addition, we showed previously that under the conditions tested N1 did not affect NF-κB activation in VACV-infected cells [33]. Therefore, B14, but not N1, associates with the IKK complex and thereby inhibits NF-κB responsive gene expression.

Concerning the site of action of B14, it is clear that B14 shuts down expression of reporter genes with NF-κB-responsive promoters in response to multiple stimuli (Figure 1) and that within infected cells the overall level of IκBα is not altered by virus infection (Figure 5) or by the expression of B14 in resting cells (Figure 4). However, in the presence of B14 there is a reduced level of phospho-IκBα in the infected cell lysates (Figure 5) and a reduced degradation of IκBα in TNFα-stimulated cells. These findings suggest a possible effect of B14 on the IKK activity. Direct evidence for the reduced phosphorylation of IκBα by IKK in the presence of B14 was provided by an in vitro kinase assay using a synthetic IκBα peptide substrate and IKK that had been immunoprecipitated from cells (Figure 6). Therefore, the mechanism of action of B14 lies upstream of IκBα phosphorylation. Consistent with this, B14 was found to co-purify with the IKK complex from infected cells and to co-precipitate with the IKK complex using specific antibodies either against tagged B14, NEMO (Figures 2 and 3), or against IKKα/β (unpublished data). Notably, the assembly of the IKK complex was not interrupted by B14. Furthermore, use of IKK null MEFs revealed that IKKβ is the target of B14 in the complex and B14 did not bind to or disrupt the IKKα-NEMO complex (Figure 3B). These findings indicate that the inhibitory effect of B14 on the activity of the IKK complex is not due to disassembly of the IKK complex.

B14 inhibited NF-κB activation driven by overexpression of either IKKα or IKKβ (Figure 7A) or by expression of the constitutively active IKKα SS/EE mutant in which the ser176 and ser180 in the activation loop were mutated to glutamic acid (Figure 7B). In contrast, B14 was unable to inhibit NF-κB gene expression by a similar constitutively active IKKβ SS/EE mutant (Figure 7B), indicating IKKβ but not IKKα is the target for B14. Furthermore, B14 associated with the IKK complex via IKKβ (Figure 3) and inhibited phosphorylation of IKKβ in the activation loop (Figure 8), thereby downregulating the activity of the IKK complex. However, once the IKKβ subunit is activated, B14 may not be inhibitory.

B14 co-purified with the IKK complex but was also present in a 160-kDa complex, much larger than the mass of monomeric B14 (17.3 kDa). Consistent with these findings, recombinant B14 made in Escherichia coli was oligomeric (unpublished data). Whether B14 is the only protein in the 160-kDa complex or whether it is complexed with other unidentified cellular or viral protein(s) is unknown. However, its presence in this complex suggests B14 might have function(s) additional to that described here. For instance, the slight increase of PMA-induced AP-1 activity in the presence of B14 (Figure 1D) may result from interaction of B14 with an unidentified protein(s). Alternatively, this may be a consequence of the downregulation of NF-κB responsive genes that negatively regulate AP-1 activity. There is ample precedent for small VACV proteins having more than one immunomodulatory activity. For instance, protein A52 is both a TLR inhibitor and an activator of p38 kinase to modulate IL-10 [48].

In summary, VACV virulence factor B14 inhibits the IKK signalosome by preventing phosphorylation of IKKβ in the activation loop, resulting in inhibition of NF-κB-dependent gene expression. This mechanism of action fits with the observed increased inflammatory response in vivo to infection with a virus lacking gene *B14R* [37]. Overall our findings reveal a novel strategy used by VACV to modulate cellular signaling pathways to aid viral immune evasion. The B14 may be an interesting target to develop anti-inflammatory therapeutics directed against the IKK complex.

## Materials and Methods

### Cell culture.

Human embryonic kidney (HEK) 293 cells (a gift from Dr. Paul Farrell, Imperial College London), wild type, IKKα null, and IKKβ null MEF cells (provided by Dr. Michael Karin, UCSD) were cultured in Dulbecco's modified Eagle's medium (DMEM, Gibco BRL) supplemented with 10% heat-treated foetal bovine serum (FBS, heat-treated at 56 °C for 30 min, Harlan Sera-Lab), 50 IU/ml penicillin and 50 μg/ml streptomycin (Gibco BRL) and 2 mM L-glutamine (Gibco BRL). HeLa cells were maintained in Minimum Essential Medium (MEM Gibco BRL) supplemented with 1 x non-essential amino acid solution (Sigma) and identical chemicals as DMEM. The cells were incubated in a humidified incubator (Heraeus) with 5% CO_2_.

### Plasmids, antibodies, and recombinant VACVs.

Expression vectors, VACV strains that do or do not express B14, and rabbit anti-serum against B14 have been described previously [37]. Plasmids expressing IKKs and IKK constitutively active mutants were kindly provided by Dr. Alain Chariot (University of Liège) and Dr. Richard Gaynor (Lilly Corporate Center), respectively. Reporter and TRAF2 plasmids were gifts from Dr. Andrew Bowie (Trinity College Dublin). Anti-IKKγ (NEMO) (BD Biosciences), anti-HA (Cambridge Biosciences), anti-GSK3β (BD Biosciences) mAbs were used for immunoprecipitation or immunoblotting. For immunoblotting, rabbit polyclonal anti-IKKα (Cell Signaling), anti-IKKα/β (Santa Cruz), NEMO (Cell Signaling), and IκBα (Santa Cruz) were used. In addition, murine mAb anti-P-IκBα (Cell Signaling), α-tubulin, IKKα and IKKβ (Upstate) were used. The anti-N1 polyclonal Ab was described previously [24]. Lastly, rabbit mAb against phospho-IKKα/β (16A6, Cell Signaling) was used to detect IKKβ that is phosphorylated at Ser177/181.

### Reporter assay.

HeLa cells (8 × 10^4^ per well) were seeded and then transfected with 100 ng of reporter plasmids, 50 ng of pSV-β-galactosidase (Promega), and the indicated amount of expression vectors with FuGENE 6 (Roche). The total amount of DNA (400 ng) was kept constant by supplementation with pCI (Promega). After overnight incubation, the transfected cells were simulated with 100 ng/ml of IL-1β, TNFα (Peprotech), or 50 ng/ml of PMA (Sigma) for 8 h. Cells were harvested in passive lysis buffer (Promega), and the relative stimulation of NF-κB activity was calculated by normalizing luciferase activity with β-galactosidase activity.

HEK 293 cells (6 × 10^4^ per well) were seeded into 24-well tissue culture plates overnight before transfection. Reporter plasmids (90 ng), 10 ng of pTK-*Renilla* luciferase (pRL-TK, a gift from Dr. Andrew Bowie), and the indicated amount of expression vectors were delivered into cells with FuGENE 6. The total amount of DNA (500 ng) was kept constant by supplementation with pCI (Promega). After 24 h, cells were harvested in passive lysis buffer (Promega), and the relative stimulation of NF-κB-dependent gene expression was calculated by normalizing luciferase activity with *Renilla* luciferase intensity. In case of stimulations, the cells were incubated with stimuli described previously and 5 μg/ml of poly (I:C) (Invitrogen) for 12 h before lysis.

In all cases, data shown are from one of three to five independent experiments with similar qualitative results. Data from experiments performed in triplicate are expressed as means ± SD.

### Immunoprecipitation and immunobloting.

HeLa cells in 10-cm dishes were infected by vB14-HA or vΔB14 (10 p.f.u. per cell). At 4 h p.i., the infected cells were washed once with ice-cold PBS and lysed with IP buffer (50 mM HEPES [pH 7.5], 100 mM NaCl, 1 mM EDTA, 10% [v/v] glycerol, 0.5%[v/v] Nonidet P-40 containing 1 mM phenylmethylsulphonyl fluoride, 0.01% [v/v] aprotinin, and 1 mM sodium orthovanadate). For immunoprecipitation, the indicated antibodies were pre-incubated with protein G-Sepharose (Amersham) at 4 °C for 1 h. Then equal amounts of the beads were added and incubated with the cell lysates overnight at 4 °C. The immune complexes were washed, boiled with 30 μl of 5 x sample buffer, and analysed by immunoblotting.

Proteins were resolved and transferred to nitrocellulose membranes (Hybond ECL, Amersham). After transfer, the membranes were rinsed once in PBS and then incubated with blocking buffer (PBS containing 5% Marvel milk powder) for 30 min at RT. The primary Ab was added to the blocking buffer and incubated for 1 h on a rocking platform. The membranes were washed five times, for 6 min, with PBS, and then HRP-conjugated secondary Ab (Sigma) was added in blocking buffer. After 45-min incubation, the membranes were washed as above and then incubated with chemiluminescence reagent (ECL, Amersham) for signal detection. The membranes were wrapped in Saran wrap and exposed to X-ray film (Kodak).

### Size exclusion chromatography.

At 4 h p.i., the infected cells were washed once with ice-cold PBS and lysed with CE buffer (10 mM 4-(2-hydroxyethyl)-1-piperazineethanesulfonic acid (HEPES), 10 mM KCl, 0.1 mM EDTA (pH 8.0), 0.1 mM EGTA, 1 mM DTT, 0.05 % NP-40, 20 mM β-glycerophosphate, 1 mM Na_3_VO_4_, 1 x protease inhibitor cocktail II (CalBio) for 30 min at 4 °C. The extract was centrifuged at 10,000 x *g* for 1 h at 4 °C. Five hundred μl of the supernatant was loaded onto a Superose 6 gel filtration column (Amersham) that had been equilibrated in gel filtration (GF) buffer (25 mM Tris-HCl [pH 7.6], 150 mM NaCl, 0.2% NP-40, 1 mM DTT). Approximately 10%–15% of each fraction was analysed by SDS-PAGE followed by immunoblotting with the indicated Ab. The column was calibrated in the GF buffer using protein standards kits from Amersham.

### In vitro kinase assay.

HEK 293 cells were transfected using the indicated vectors overnight and lysed in lysis buffer (20 mM Tris [pH 7.4], 150 mM NaCl, 1 mM EDTA, 1 mM EGTA, 1% Triton X-100, 2.5 mM sodium pyrophosphate, 1 mM β-glycerophosphate, 1 mM Na_3_VO_4_, 1 μg/ml leupeptin, and 1 mM phenylmethylsulfonyl fluoride). The HA-tagged IKK proteins in the cell lysate were immunoprecipitated using anti-HA mAb. The precipitate was washed three times in lysis buffer and twice in kinase buffer (20 mM Hepes/KOH [pH 7,4], 25 mM β-glycerophosphate, 2 mM dithiothreitol, 20 mM MgCl_2_). The kinase assay was performed in a final volume of 20 μl of kinase buffer containing 10 μM ATP, 5 μCi of [γ-^32^P] ATP and 1 μg of IKK substrate peptide (Upstate) derived from IκBα sequence (KKKKERLLDDRHDSGLDSMKDEE). After incubation for 10 min at 30 °C, the reaction was stopped by the addition of 5× SDS sample buffer. Proteins were separated by SDS-PAGE and stained by Coomasie blue. ^32^P-labelled proteins were visualized by autoradiography.

### LUMIER.

For LUMIER assays [42], 293 ET cells were transfected with a pair of putative interactors fused to Renilla luciferase or HA/FLAG antibody tags. Post-nuclear supernatants from cells lysed in IP buffer (10% glycerol, 150 mM NaCl, 20 mM Tris-HCl [pH 7.4], 0.1 % Triton-X100, and inhibitors) were incubated with HA or FLAG agarose (Sigma). After washing, proteins were eluted for 30 min with 150 μg/ml FLAG peptide or 100 μg/ml HA peptide in Renilla lysis buffer (Promega). The ratio between luciferase activity in eluates and lysates is presented as fold binding over a control reaction.
